# Adaptive Phase Rolling for Opportunistic Beamforming in OFDMA Systems with a Small Number of Users

**DOI:** 10.1155/2014/625421

**Published:** 2014-04-14

**Authors:** Minjoong Rim

**Affiliations:** Department of Information and Communication Engineering, Dongguk University, 30 Pildong-ro 1 Gil, Jung-gu, Seoul 100-715, Republic of Korea

## Abstract

The performance of opportunistic beamforming might be degraded if the number of users is small. This paper proposes an adaptive opportunistic beamforming technique for orthogonal frequency division multiple access systems, which can produce good results even with a small number of users. This paper also proposes a modified proportional fairness scheduling algorithm, which can further improve the performance of the proposed opportunistic beamforming technique.

## 1. Introduction

The throughput of a wireless communication system can be improved using scheduling if the number of users is large and the channels are sufficiently time-selective. A channel-aware scheduling technique tries to select and allocate a user with a good channel condition and the multiuser diversity effect is maximized with a large number of candidates for scheduling [1, 2]. If wireless channels are not time-varying, an opportunistic beamforming technique with random beamforming or phase rolling (phase rotations) can be applied to make the channels time-selective [3–10]. Opportunistic beamforming can be achieved by applying different random beamforming weights each time slot. However, for some cases, such discontinuous changes of channel states may degrade the performance of channel estimation or channel quality estimation. Continuous changes of the channel states can be implemented by multiplying continuously rotating phases to signals for some antennas. This paper discusses modifications to phase rolling techniques for improving the system throughputs.

Although an opportunistic beamforming scheme needs only the channel quality information (CQI), it can produce a performance comparable to a transmit beamforming method, which requires the channel state information (CSI) [3–10]. However, the performance of an opportunistic beamforming technique can be degraded if the number of users is small since there will be too few users participating in scheduling and none of the candidate channels might be satisfactory. Several methods are proposed to improve the performance even with a small number of users [11–14]. In some methods, appropriate beamforming patterns are found from the previous time slots assuming that the channels vary very slowly [11, 12]. Such methods may require a large memory and produce good results only when the channels change slowly. In some other schemes, multiple minislots with different random beamforming are used for each time slot to generate multiple different channels and select the weighting factor with the largest channel quality for data transmissions [13, 14]. Although these schemes can produce good results, substantial modifications to the air interface may be required.

While these methods are based on random beamforming, this paper proposes a simple opportunistic beamforming technique based on phase rolling for orthogonal frequency division multiple access (OFDMA) systems. The proposed technique is similar to conventional phase rolling schemes but adaptively changes the direction of phase rotations to produce satisfactory results even with a small number of users. This paper also proposes a modification to channel-aware scheduling algorithms to further improve the performance of the proposed opportunistic beamforming technique.

The rest of this paper is organized as follows.  Section 2 describes the system model considered in this paper.  Section 3 describes conventional methods including a transmit beamforming scheme, a phase rolling technique, and a proportional fairness scheduling algorithm. In Section 4, an adaptive phase rolling technique is proposed for improving the performance. Also, modification to the proportional fairness scheduling algorithm is presented. Simulation results are shown in Section 5 and conclusions are drawn in Section 6.

## 2. System Model 

**Figure 1:**
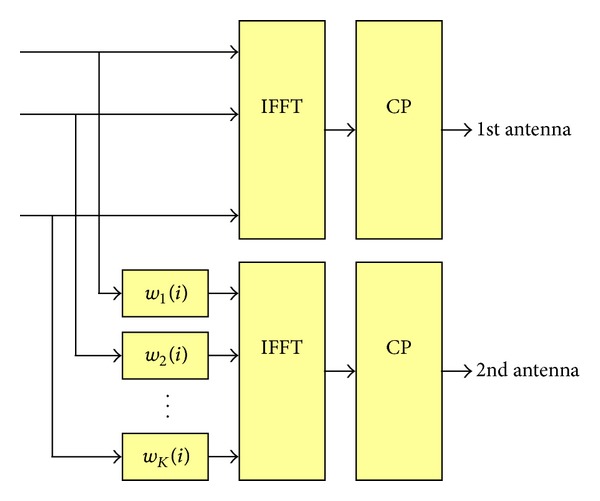
OFDMA system with phase rolling.

Suppose that a resource block (RB) of a downlink OFDMA system is composed of two-dimensional resource elements of *N*_Subcarrier_ subcarriers and *N*_Symbol_ OFDM symbols (a time duration of *N*_Symbol_ OFDM symbols is called a time slot in this paper), and each RB includes dedicated pilots to obtain the CQI or CSI for the RB. The transmitter (base station) receives the CQI from *M* receivers (mobile stations) every time slot and performs scheduling with *K* RBs. This paper considers an OFDMA system with two transmit antennas with equal transmit power and one receive antenna per receiver. Although the proposed schemes as well as the conventional schemes can be extended to more than two antennas, only two transmit antennas are considered in this paper for simplicity of discussion. As shown in Figure 1, an opportunistic beamforming technique can be implemented by multiplying a time-varying weighting factor to each RB at the second antenna.

Let RB_*k*_(*i*) be the *k*th RB at the *i*th time slot. Assuming a time-flat and frequency-flat fading channel over a single RB, the received signal *r*_*m*,*k*,*p*,*q*_(*i*) at the *q*th subcarrier of the *p*th OFDM symbol in RB_*k*_(*i*) for the *m*th user can be written as

(1) rm,k,p,q(i)=h~m,k(i)xm,k,p,q(i)+nm,k,p,q(i),

where *x*_*m*,*k*,*p*,*q*_(*i*) is the transmitted signal, *w*_*k*_(*i*)(|*w*_*k*_(*i*)| = 1) is the weighting factor for the second antenna, *n*_*m*,*k*,*p*,*q*_(*i*) is the corresponding noise, and the resulting channel ${\tilde{h}}_{m,k}(i)$ is

(2) $$\begin{array}{c} {\tilde{h}}_{m,k}\left(i\right)=h_{1,m,k}\left(i\right)+w_{k}\left(i\right)h_{2,m,k}\left(i\right), \end{array}$$

where *h*_1,*m*,*k*_(*i*) and *h*_2,*m*,*k*_(*i*) are the channels for the two antennas. The data rate of the *m*th user can be determined by the resulting channel energy, written as

(3) |h~m,k(i)|2=|h1,m,k(i)+wk(i)h2,m,k(i)|2=|h1,m,k(i)|2+|h2,m,k(i)|2 +2|h1,m,k(i)||h2,m,k(i)|cos⁡(Θm,k(i)),

where Θ_*m*,*k*_(*i*) is the phase difference between the two channels *w*_*k*_(*i*)*h*_2,*m*,*k*_(*i*) and *h*_1,*m*,*k*_(*i*), expressed as

(4) $$\begin{array}{c} \Theta_{m,k}\left(i\right)\equiv \mathrm{mod}\left(∠h_{2,m,k}\left(i\right)+∠w_{k}\left(i\right)-∠h_{1,m,k}\left(i\right),2\pi \right). \end{array}$$

In (4), mod⁡ (*ϕ*, 2*π*) is the modulo operation, representing a phase *ϕ* as a value between 0 and 2*π*, written as

(5) $$\begin{array}{c} \mathrm{mod}\left(\phi ,2\pi \right)\equiv \phi -\left\lfloor \frac{\phi }{2\pi }\right\rfloor 2\pi , \end{array}$$

where ⌊•⌋ is the floor operation. The energy of the resulting channel, defined as ${\left|{\tilde{h}}_{m,k}^{}(i)\right|}^{2}$ in this paper, is maximized as

(6) $$\begin{array}{c} {\left|{\tilde{h}}_{m,k}^{}(i)\right|}^{2}={\left(\left|h_{1,m,k}(i)\right|+\left|h_{2,m,k}(i)\right|\right)}^{2} \end{array}$$

when Θ_*m*,*k*_(*i*) is near 0 or 2*π* and minimized as

(7) $$\begin{array}{c} {\left|{\tilde{h}}_{m,k}^{}(i)\right|}^{2}={\left(\left|h_{1,m,k}(i)\right|-\left|h_{2,m,k}(i)\right|\right)}^{2} \end{array}$$

when Θ_*m*,*k*_(*i*) is around *π*.

## 3. Conventional Methods 

### 3.1. Beamforming

If the *m*th user is assigned to RB_*k*_(*i*) and the CSI is available at the transmitter, a beamforming technique can be applied and the weighting factor can be written as follows:

(8) $$\begin{array}{c} w_{k}^{\text{Beamforming}\;}\left(i\right)=\exp\left(j\left(∠h_{1,m,k}\left(i-1\right)-∠h_{2,m,k}^{}\left(i-1\right)\right)\right). \end{array}$$

Suppose that the channels are changing very slowly; in other words, *h*_1,*m*,*k*_(*i* − 1) ≈ *h*_1,*m*,*k*_(*i*) and *h*_2,*m*,*k*_(*i* − 1) ≈ *h*_2,*m*,*k*_(*i*). Then, the phase difference is written as

(9) Θm,k(i)=mod⁡(∠h2,m,k(i)−∠h1,m,k(i) +∠h1,m,k(i−1)−∠h2,m,k(i−1),2π)≈0  or  2π,

and the channel energy can be maximized. However, using the CSI at the transmitter requires substantial amount of feedback information from the receivers and may not be practical for some cases.

### 3.2. Phase Rolling

**Figure 2:**
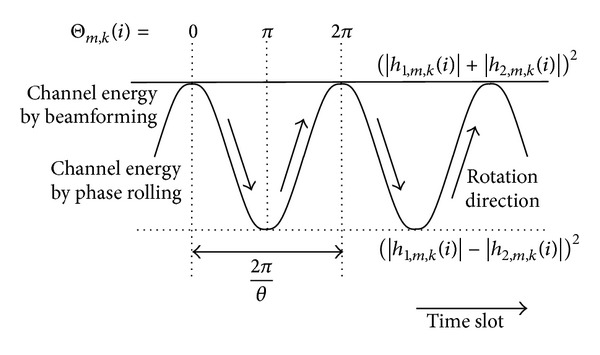
Channel energy by phase rolling.

If the CSI is unknown but the CQI is available at the transmitter, multiuser diversity can be exploited by making the channel sufficiently time-selective using time-varying weighting factors [3, 4]. When a phase rolling technique is applied, the weighting factor *w*_*k*_(*i*) for RB_*k*_(*i*) can be written as

(10) $$\begin{array}{c} w_{k}^{\text{PhaseRolling}}\left(i\right)=w_{k}^{\text{PhaseRolling}}\left(i-1\right)\exp\left(j\theta \right), \end{array}$$

where *j* ^2^ = −1, *θ* determines the speed of the phase rotation, and the initial weighting factor *w*_*k*_(0) can be randomly chosen. Suppose that *θ* is very small compared to 2*π* and the channels for the *m*th user are changing very slowly and thus *h*_1,*m*,*k*_(*i* + *n*) ≈ *h*_1,*m*,*k*_(*i*) and *h*_2,*m*,*k*_(*i* + *n*) ≈ *h*_2,*m*,*k*_(*i*) for *n* = 1,…, [2*π*/*θ*], where [•] is the rounding operation finding the nearest integer. The resulting phase difference for the *m*th user at the (*i* + *n*)th time slot can be written as

(11) $$\begin{array}{c} \Theta_{m,k}^{}\left(i+n\right)\approx \mathrm{mod}\left(\Theta_{m,k}^{}\left(i\right)+n\theta ,2\pi \right) \end{array}$$

for *n* = 1,…, [2*π*/*θ*]. The phase difference in (11) is continuously changing and the resulting channel in (3) is fluctuating in time as shown in Figure 2. The phase difference becomes near 0 or 2*π* and the resulting channel energy is maximized when *n* = *n*_max⁡_, where

(12) $$\begin{array}{c} n_{\max}=\left[\frac{\mathrm{mod}\left(2\pi -\Theta_{m,k}\left(i\right),2\pi \right)}{\theta }\right]. \end{array}$$

Similarly, the phase difference becomes near *π* and the resulting channel energy is minimized when *n* = *n*_min⁡_, where

(13) $$\begin{array}{c} n_{\min}=\left[\frac{\mathrm{mod}\left(\pi -\Theta_{m,k}\left(i\right),2\pi \right)}{\theta }\right]. \end{array}$$

If scheduling can be performed on time slots with good resulting channels most of the time, the performance of the phase rolling technique can be comparable to that of the beamforming.

### 3.3. Proportional Fairness Scheduling

One of the most popular scheduling methods for wireless communications is the proportional fairness (PF) algorithm [1, 2]. Using the PF algorithm, the scheduled user *S*_*k*_(*i*) for RB_*k*_(*i*) can be determined as

(14) Sk(i)=argmaxmDm,k(i)Rm(i−1)Rm(i)=(1−1T)Rm(i−1)+1T∑k=1Mδm,k(i)Dm,k(i),

where *D*_*m*,*k*_(*i*) is the amount of data possibly transmitted to the *m*th user through RB_*k*_(*i*) obtained by the CQI feedback, *R*_*m*_(*i*) is the amount of recently received data by the *m*th user until the *i*th time slot, *T* is the window size to calculate *R*_*m*_(*i*), and *δ*_*m*,*k*_(*i*) is defined as

(15) $$\begin{array}{c} \delta_{m,k}\left(i\right)\equiv \{\begin{array}{cc} 1 & \text{if}\;S_{k}\left(i\right)=m,\\ 0 & \text{otherwise}. \end{array} \end{array}$$

Using the PF scheduling algorithm, the data amount actually transmitted through RB_*k*_(*i*), denoted as ${\overset{-}{D}}_{k}(i)$, is written as

(16) $$\begin{array}{c} {\overset{-}{D}}_{k}\left(i\right)=D_{S_{k}(i),k}\left(i\right). \end{array}$$

Assuming that a data amount transmitted through an RB is proportional to the corresponding channel energy, in other words,

(17) $$\begin{array}{c} D_{m,k}\left(i\right)\propto {\left|{\tilde{h}}_{m,k}\left(i\right)\right|}^{2}, \end{array}$$

there is a greater chance of scheduling when the channel energy becomes good. Since the PF scheduling with the phase rolling scheme selects near-best users among *M* users with random beamforming, its performance can be comparable to that of the transmit beamforming technique when the number of users *M* is very large. However, the scheduling performance might be degraded if the number of users is small since the choice needs to be made among a small number of candidates.

## 4. Proposed Methods

### 4.1. Adaptive Phase Rolling

When the number of users is small, a user scheduled on an RB may have a greater chance to be scheduled on the same RB at the next time slot. If the same user is assigned to an RB for consecutive time slots, the phase rotation direction for the RB can be chosen to improve the performance of the user expecting that it might be scheduled on the same RB again at the next time slot. In the proposed adaptive phase rolling technique, the phase rotation direction for an RB is altered if the same user is scheduled on the RB for consecutive time slots and the data amount transmitted through the RB is decreased. Otherwise, it remains unchanged. Let *d*_*k*_(*i*) (= 1 or −1) be the phase rotation direction for RB_*k*_(*i*). In the proposed phase rolling technique, the weighting factor *w*_*k*_(*i*) for RB_*k*_(*i*) can be written as

(18) wkProposed(i)=wkProposed(i−1)exp⁡(jdk(i−1)θ),dk(i)={−dk(i−1)if  Sk(i)=Sk(i−1),  D−k(i)<D−k(i−1)dk(i−1)otherwise,

where the initial value of the phase rotation direction *d*_*k*_(0) is 1. Suppose that the same user is assigned to an RB over multiple consecutive time slots, the phase rotation direction is chosen to improve the performance, and the weighting factor in (18) will be updated toward the beamforming weighting factor in (8). If different users are scheduled on an RB every time, (18) becomes (10), resulting in the conventional phase rolling. The proposed method provides the multiuser diversity effect when the number of users is large and attempts to obtain the beamforming effect with a small number of users.

Suppose that *θ* is very small compared to 2*π*; the *m*th user is repeatedly assigned to the *k*th RB, and the channels are varying very slowly and thus *h*_1,*m*,*k*_(*i* + *n*) ≈ *h*_1,*m*,*k*_(*i*) and *h*_2,*m*,*k*_(*i* + *n*) ≈ *h*_2,*m*,*k*_(*i*) for *n* = 1,…, [2*π*/*θ*]. The phase difference for the *m*th user at the (*i* + 1)th time slot can be represented as

(19) $$\begin{array}{c} \Theta_{m,k}^{}\left(i+1\right)\approx \mathrm{mod}\left(\Theta_{m,k}\left(i\right)+d_{k}\left(i\right)\theta ,2\pi \right) \end{array}$$

and the difference between the channel energy values ${\left|{\tilde{h}}_{m,k}(i+1)\right|}^{2}$ and ${\left|{\tilde{h}}_{m,k}(i)\right|}^{2}$ can be written as follows:

(20) |h~m,k(i+1)|2−|h~m,k(i)|2≈2|h1,m,k(i)||h2,m,k(i)| ×{cos⁡(Θm,k(i)+dk(i)θ)−cos⁡(Θm,k(i))}.

Assuming that a data amount transmitted through an RB is proportional to the corresponding channel energy, the rotation direction *d*_*k*_(*i* + 1) is determined as

(21) when  0<Φm,k(i)<πdk(i+1)={−dk(i)if  dk(i)=1dk(i)otherwise,when  π<Φm,k(i)<2πdk(i+1)={dk(i)if  dk(i)=1−dk(i)otherwise,when  Φm,k(i)=0  or  πdk(i+1)=dk(i),

where

(22) $$\begin{array}{c} \Phi_{m,k}\left(i\right)=\mathrm{mod}\left(\Theta_{m,k}\left(i\right)+d_{k}\left(i\right)\frac{\theta }{2},2\pi \right). \end{array}$$

Equation (21) can be simplified as

(23) dk(i+1)={−1if  0<Φm,k(i)<π1if  π<Φm,k(i)<2πdk(i)if  Φm,k(i)=0  or  π.

**Figure 3:**
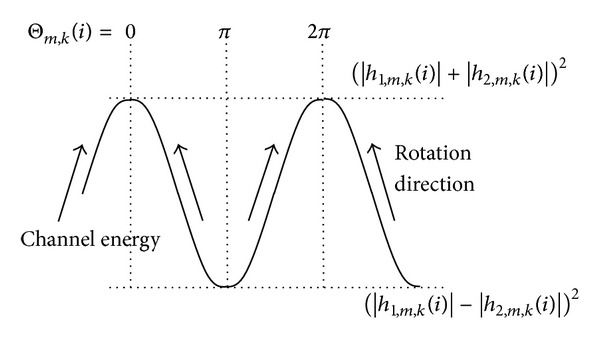
Rotation directions by adaptive phase rolling.

As shown in Figure 3, the rotation direction in the adaptive phase rolling scheme is chosen to increase the channel energy.

If ${\left|{\tilde{h}}_{m,k}(i+1)\right|}^{2}<{\left|{\tilde{h}}_{m,k}(i)\right|}^{2}$ and the rotation direction *d*_*k*_(*i* + 1) at the (*i* + 1)th time slot is changed to −*d*_*k*_(*i*), then

(24) Θm,k(i+2) ≈mod⁡(Θm,k(i+1)+dk(i+1)θ,2π) ≈mod⁡(Θm,k(i)+dk(i)θ+dk(i+1)θ,2π) =Θm,k(i)

and thus

(25) |h~m,k(i+2)|2−|h~m,k(i+1)|2≈|h~m,k(i)|2−|h~m,k(i+1)|2<0.

Hence, the rotation direction at the (*i* + 2)th time slot remains unchanged; in other words, *d*_*k*_(*i* + 2) = *d*_*k*_(*i* + 1). If ${\left|{\tilde{h}}_{m,k}(i+1)\right|}^{2}\geq {\left|{\tilde{h}}_{m,k}(i)\right|}^{2}$ and the rotation direction *d*_*k*_(*i* + 1) remains as *d*_*k*_(*i*), the direction *d*_*k*_(*i* + *n*) will be unchanged while 1 ≤ *n* ≤ *n*_max⁡_, where

(26) $$\begin{array}{c} n_{\max}=\left[\frac{\mathrm{mod}\left(2\pi -d_{k}\left(i\right)\Theta_{m,k}\left(i\right),2\pi \right)}{\theta }\right] \end{array}$$

and the phase difference can be written as

(27) $$\begin{array}{c} \Theta_{m,k}^{}\left(i+n\right)\approx \mathrm{mod}\left(\Theta_{m,k}\left(i\right)+nd_{k}\left(i\right)\theta ,2\pi \right) \end{array}$$

for 1 ≤ *n* ≤ *n*_max⁡_ + 1. Since

(28) nmax⁡dk(i)θ =[mod⁡(2π−dk(i)Θm,k(i),2π)θ]dk(i)θ ≈{2π−Θm,k(i)if  dk(i)=1,Θm,k(i)≠0−Θm,k(i)otherwise

for a small *θ*, the phase difference at the (*n*_max⁡_)th time slot is written as

(29) Θm,k(i+nmax⁡)≈mod⁡(Θm,k(i)+nmax⁡dk(i)θ,2π)≈0  or  2π

and the channel energy is maximized. Similarly, the phase difference at the (*n*_max⁡_ + 1)th time slot can be approximated as

(30) Θm,k(i+nmax⁡+1) ≈mod⁡(Θm,k(i)+nmax⁡dk(i)θ+dk(i)θ,2π) ≈θ  or  2π−θ

and the rotation direction is altered; in other words, *d*_*k*_(*i* + *n*_max⁡_ + 1) = −*d*_*k*_(*i* + *n*_max⁡_). After the channel energy reaches the maximum, the rotation direction is repeatedly altered so that the phase difference between the two channels remains very small. Hence, the resulting channel energy will stay around the maximum point, producing system performance close to that obtained by the beamforming in (8).

### 4.2. Modified Proportional Fairness Scheduling

The proposed method in (18) is useful when the same user is assigned to an RB over multiple consecutive time slots. In order to further improve the performance of the proposed technique, the PF algorithm in (14) can be modified. There can be many methods to achieve this and one simple modification to the PF algorithm is, for each RB, to give a slightly higher priority to the user scheduled on the RB in the previous time slot. In the modified PF algorithm, the scheduled user *S*_*k*_^Modified  PF^(*i*) for RB_*k*_(*i*) can be determined as

(31) $$\begin{array}{c} S_{k}^{\text{Modifed}\;\text{PF}}\left(i\right)=\underset{m}{\text{argmax}}\left(1+\alpha \;\delta_{m,k}\left(i-1\right)\right)\frac{D_{m,k}\left(i\right)}{R_{m}\left(i-1\right)}, \end{array}$$

where *α* is the weighting factor to give a higher priority to user *S*_*k*_^Modified  PF^(*i* − 1).

## 5. Simulation Results

In this section, the transmit beamforming in (8), conventional phase rolling in (10), and proposed adaptive phase rolling in (18) techniques are compared with the PF in (14) and modified PF scheduling algorithms in (31). Simulations are performed with 16 RBs and varying the number of users from 1 to 24. Equal power allocation to the antennas is assumed and the delay from CQI estimation to actual scheduling is ignored. The detailed simulation parameters are shown in Table 1.

**Figure 4:**
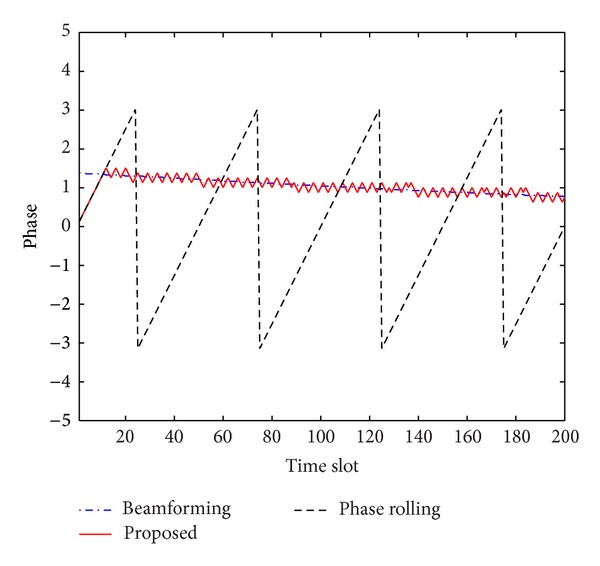
Phase of the second antenna.

**Figure 5:**
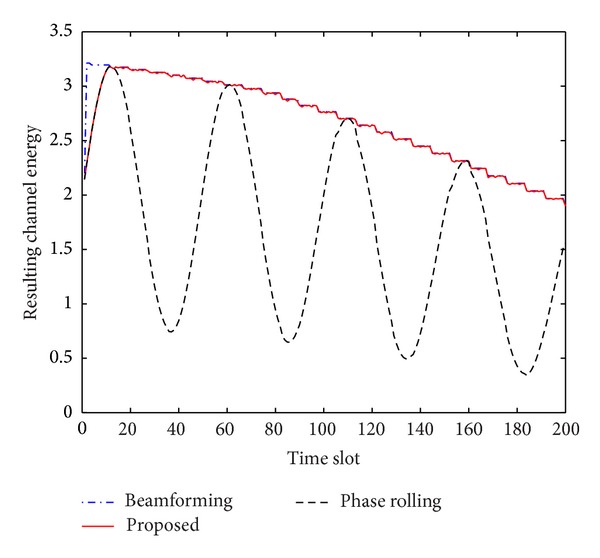
Resulting channel energy.

Figure 4 illustrates the phase trajectories of the second antenna with a single user. Only the first RB is monitored. While the phase of the second antenna is moved to one direction by the conventional phase rolling technique, the proposed scheme adaptively changes the phase rotation direction and can produce near-optimal phases after initialization of a few time slots.  Figure 5 represents the resulting channel energy produced by the beamforming, conventional phase rolling, and proposed techniques. While the conventional phase rolling technique makes the resulting channel fluctuate, the proposed scheme produces results similar to those by the transmit beamforming technique.

**Figure 6:**
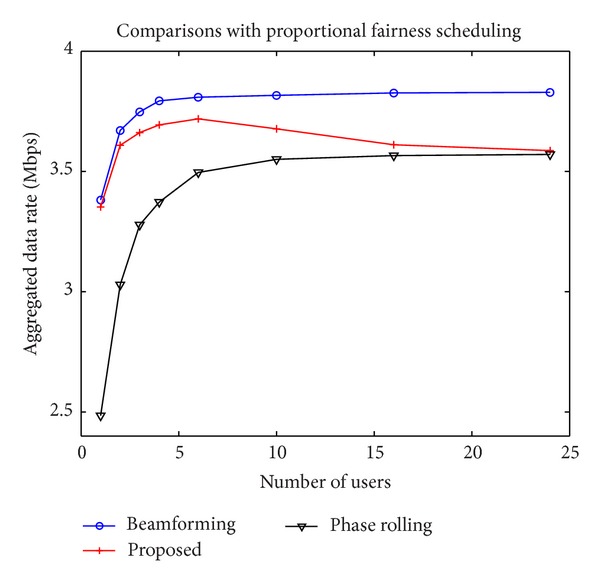
Data rates with the PF scheduling algorithm.

**Figure 7:**
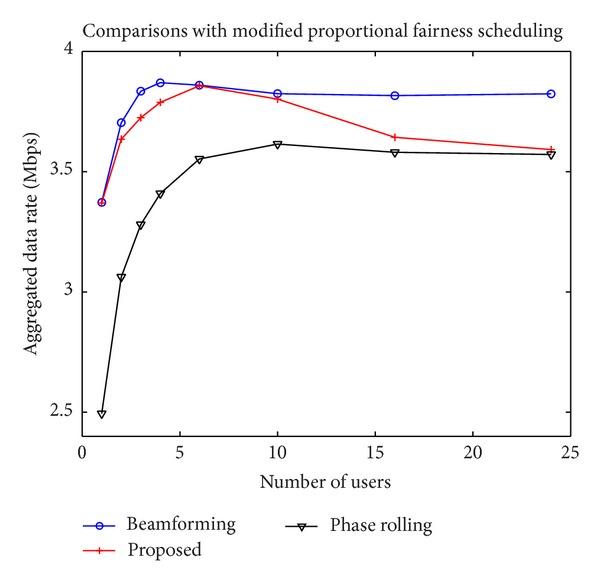
Data rates with the modified PF scheduling algorithm.

Figure 6 illustrates the aggregated data rates with the PF scheduling algorithm. The proposed technique performs similarly to the conventional phase rolling technique when the number of users is large and close to the transmit beamforming with a small number of users. The conventional phase rolling technique produces good results only when the number of users is large but the proposed technique is useful regardless of the number of users.  Figure 7 shows the aggregated data rates with the modified PF scheduling algorithm. The improvement of the proposed technique using the modified PF algorithm is noticeable since the proposed technique is effective when the same user is allocated to an RB over multiple consecutive time slots.

## 6. Conclusion

When scheduling is performed with RBs in OFDMA systems, an opportunistic beamforming technique with a phase rolling technique can make the channels time-selective and produce good results with a large number of users. However, its performance may be degraded with a small number of users since there are insufficient candidates for scheduling. This paper proposes a modified phase rolling technique, whose phase rolling direction is changed adaptively to achieve the beamforming effect especially when the number of users is small. The performance improvement of the proposed technique is noticeable especially with the modified PF algorithm, where there is a greater chance that the same user is assigned to an RB over multiple consecutive time slots.

## Figures and Tables

**Table 1:** Simulation parameters.

Parameters	Values
Sampling frequency	1 MHz
FFT size	128
Cyclic prefix length	16 samples
Number of users (*M*)	1~24
Number of resource blocks (*K*)	16
Number of OFDM symbols in RB (*N*_Symbol_)	1
Number of subcarriers in RB (*N*_Subcarrier_)	8
Carrier frequency	2 GHz
Mobility of users	3 Km/h
Channel power-delay-profile distribution	Exponential
Root-mean-square delay spread	0.67 *μ*s
Channel signal-to-noise ratio	10 dB
Phase rotation speed (*θ*)	2*π* × 0.02
Scheduling algorithm	Proportional fairness
Window size (*T*)	4
Weighting factor (*α*)	0.1
